# Changes in COPD-related anxiety symptoms during pulmonary rehabilitation: a prospective quantitative and qualitative study

**DOI:** 10.3389/fresc.2024.1428893

**Published:** 2024-08-07

**Authors:** Ingeborg Farver-Vestergaard, Eva Holmegaard Buksted, Dorthe Sørensen, Sune Jonstrup, Henrik Hansen, Camilla Fischer Christiansen, Anders Løkke

**Affiliations:** ^1^ Department of Medicine, Lillebaelt Hospital, Vejle, Denmark; ^2^ Department of Regional Health Research, University of Southern Denmark, Odense, Denmark; ^3^ VIA Research Centre for Health and Welfare Technology, Program for Rehabilitation, VIA University College, Aarhus, Denmark; ^4^ Department of Health, Vejle Municipality, Vejle, Denmark; ^5^ Respiratory Research Unit and Department of Respiratory Medicine, Copenhagen University Hospital, Hvidovre, Denmark; ^6^ Institute of Rehabilitation Sciences, University of Antwerp, Antwerp, Belgium

**Keywords:** chronic obstructive pulmonary disease, psychological distress, health-related quality of life, patient activation, disease management, rehabilitation

## Abstract

**Background:**

Fear-avoidance in COPD can have detrimental effects on pulmonary rehabilitation (PR) outcomes and is therefore important to address. This prospective study examined changes in and management of COPD-related anxiety symptoms over the course of a PR program.

**Methods:**

Patients with COPD referred to 9-weeks of PR in the municipality of Vejle, Denmark from January to December 2022 completed a six-minute walk test (6MWT) and the following questionnaires, both before and after PR: COPD Anxiety Questionnaire 20-item version (CAF-R), measuring COPD-related anxiety; COPD Assessment Test (CAT), measuring COPD-related disability; 12-Item Short-Form Health Survey (SF-12), measuring health-related quality of life (HR-QoL); sociodemographic and disease-related information. After PR, a subsample of the patients took part in semi-structured interviews exploring their understanding of how they managed COPD-related anxiety during PR. Pre- and post-assessment of COPD-related anxiety and other PR outcomes were analysed with *t*-tests and correlation analyses. Qualitative interviews were analysed using a thematic analysis approach.

**Results:**

A total of 72 patients with COPD (mean ± SD age 71 ± 8, 53% female) were included in the study, and 13 took part in qualitative interviews. A significant decrease in COPD-related anxiety was observed from before to after PR, corresponding to a small effect size (Cohen's *d *= 0.32; *p *= 0.018). Reductions in COPD-related anxiety were not associated with improvements in COPD-related disability, HR-QOL, or functional exercise capacity. The qualitative findings identified four anxiety management strategies, i.e., “planning”, “problem-solving”, “accepting”, and “confronting”, which were influenced by interactions with healthcare professionals and co-patients as well as patients’ own perception.

**Conclusions:**

COPD-related anxiety symptoms was reduced after PR, potentially through the use of various management strategies. The strategies appeared to be influenced by interactional factors during the PR program.

## 1 Introduction

Patients with chronic obstructive pulmonary disease (COPD) experience significant functional impairment linked to disease-related physical symptoms, e.g., shortness of breath, coughing, and airway mucus production as well as symptoms of anxiety and depression that often accompany the condition (1). In most cases, pharmacological treatment cannot fully remedy the symptoms, and multi-component pulmonary rehabilitation (PR) therefore stands as a cornerstone in the treatment of COPD (2).

PR has been defined as “*a comprehensive intervention based on a thorough patient assessment followed by patient-tailored therapies that include, but are not limited to, exercise training, education, and behavior change, designed to improve the physical and psychological condition of people with chronic respiratory disease and to promote the long-term adherence to health-enhancing behaviors*” (3). PR programs vary according to local funding and resources but are typically outpatient-based and delivered over a period of 8–12 weeks with 2–3 weekly sessions (4, 5). Generally, although these programs have shown good efficacy in improving patients’ exercise capacity, respiratory symptoms and quality of life (6), there is substantial variability in response among patients (7). This variability is believed to be attributable, in part, to the inhibiting effect of anxiety-related issues on exercise and activities of daily living (8). Anxiety, depression and stress management represent a topic that is often included in the educational elements of PR (9). However, such education is typically delivered in lectures and written information, and health literacy and individual information needs are not often taken into consideration (9). Furthermore, it is highly questionable if anxiety management delivered in education sessions can be translated into actions that can be applied in practice (10).

Clinically relevant levels of anxiety symptoms are highly prevalent in patients with COPD entering PR (11). Anxiety is often measured as a general trait [e.g., the Spielberger Anxiety Inventory (12)] or a pathological condition [e.g., the General Anxiety Questionnaire (13)]. However, measures of disease-related fears, such as the Breathlessness Catastrophizing Scale (14) and the COPD Anxiety Questionnaire (15, 16) have been developed. COPD-related anxiety may be closely related to critical processes in rehabilitation (e.g., fear-based avoidance of exertional dyspnea during physical exercise) and have been shown to be predictive of certain PR outcomes beyond general anxiety measures (17, 18). A recent systematic review of qualitative studies on COPD-related anxiety from the patient perspective showed that this type of anxiety can be maintained by internal factors, such as patients’ thoughts about symptom progression and dying, and external factors, such as reactions from those around them, witnessing other patients’ suffering, and being in an environment where they do not feel safe (19). Several of these factors come directly into play during PR, when patients are exposed to physical activity, shortness of breath during exercise, and witnessing co-patients’ disease state. However, the role of these complex experiences is unclear.

A longitudinal study showed that a relatively large proportion of patients with COPD (20.5%) had the physical capacity to stay active after PR, but did not translate this capacity into physical activity (“Can do, don't do”) (8). Factors that significantly discriminated this subgroup from the subgroup of patients, who had the physical capacity and *did* stay active after PR (“Can do, do do”), were fear-avoidance behaviors and affect regulation related to physical activity. These findings underscore the important role of anxiety management in PR, but further research is needed for a more profound understanding of COPD-related anxiety during PR.

Based on this, the aim of the present study was to introduce systematic assessment of COPD-related anxiety in a PR setting, and to explore: (1) changes in COPD-related anxiety and conventional PR outcomes over the course of 9-weeks of PR, (2) the association between changes in COPD-related anxiety and other PR outcomes, and (3) patients’ experiences with anxiety management during PR.

## 2 Methods

This prospective study was conducted at a community-based health center in Vejle, Denmark. All patients provided written and verbal informed consent. The study complied with the General Data Protection Regulation and Danish law. According to Danish legislation (the Act on Research Ethics Review of Health Research Projects § 14, Sect. 2), studies that collect data exclusively via questionnaires and/or interviews do not need approval from an ethics committee. The management at the health center approved study procedures.

### 2.1 Participants and procedures

Patients with COPD, who attended PR in between January and December 2022, were included in the study. Patients were eligible for enrollment if they (1) had a diagnosis of COPD confirmed by a physician at a hospital and/or in general practice and if they (2) were physically capable of taking part in the exercise component of PR. Exclusion criteria were (1) unstable coronary heart disease and (2) inability to speak and/or understand Danish.

Prior to enrollment, patients underwent standardized assessment, including a questionnaire package and a 6-minute walk test. These assessments were repeated after completing PR.

#### 2.1.1 The PR program

The PR program was delivered over a period of nine weeks in total; it included a 2-hour introductory session within the first week, followed by eight weeks of two weekly sessions of three hours each. The 3-hour sessions included 90 min of group-based disease-specific patient education, a 30-minute break, and 60 min of physical exercise. The educational sessions covered: COPD, medication, diet, positive expiratory pressure (PEP) devices, sleep, breathlessness and anxiety, intimacy and relationships, and daily exercise habits. The program followed the American Thoracic Society and European Respiratory Society guidelines (3). The PR team consisted of two physiotherapists, a nurse, a dietitian, and a psychologist. Health care professionals (HCPs) delivering the program had been trained in using the COPD Anxiety Questionnaire 20-item version (COPD Angstfragebog, CAF-R) to assess COPD-related anxiety during two whole-day workshops led by authors IFV and AL. They used the results of the assessment to identify and address individual, disease-related anxiety issues that could potentially act as barriers to achieving the optimal outcome of the course.

#### 2.1.2 Measurement of PR outcomes

The COPD Anxiety Questionnaire 20-item version (CAF-R) was applied to measure COPD-related anxiety (15). The instrument consists of 20 items, yielding a total score from 0 (low COPD-related anxiety) to 80 (high COPD-related anxiety). The scale shows acceptable internal consistency, with Cronbach's α value ranging from 0.77 to 0.89 (15, 16). A minimal clinically important difference (MCID) for the scale has not yet been determined.

The COPD Assessment Test (CAT) consists of eight items and was used to measure COPD-related disability, with a total score from 0 (low disability) to 40 (high disability) (20). The scale has shown good internal consistency in the population (α=0.88). A reduction of two points or more on the scale was considered a clinically meaningful change for this study (21).

The 12-Item Short-Form Health Survey (SF-12) was used to measure health-related quality of life (HR-QoL), by addressing various aspects of emotional states and daily activities (22). Total scores for the physical component score (PCS) and the mental component score (MCS), respectively, are calculated based on population norms, and range from 0 (poor health) to 100 (good health). The SF-12 has been used in various populations and demonstrates good sensitivity to change and discriminative values in COPD (23). MCIDs of 3.29 for the PCS and 3.77 for the MCS have been presented for the SF-12—although in patients with low back pain (24).

Functional exercise capacity was tested using the 6-minute walk test (6MWT). The test is commonly applied in COPD and yields a test result of 6-minute walk distance (6MWD) in meters with a MCID of 30 meters (25).

Additionally, patients’ sociodemographic and disease-related informations were recorded at baseline.

#### 2.1.3 Qualitative interviews

A subsample of 13 patients was interviewed after completion of PR. Interviews explored the subjective understanding of how COPD-related anxiety changed during PR. Patients were purposively selected for interview by author SJ, who was one of the physiotherapists delivering the program, with the aim of including patients across age, gender, degree of illness and baseline levels of COPD-related anxiety. Thirteen individual interviews were conducted either in their own home (*n* = 10) or at the Health Center (*n* = 3), at the patients’ request. Two patients were interviewed twice, as they had not completed the PR program at the time of the first interview. The exact sample size for qualitative interviews was not projected *a priori*. We continued to include participants until data saturation was reached, i.e., no new perspectives were identified in subsequent interviews (26).

Semi-structured interviews were conducted by author EHB based on an interview guide that consisted of four parts: (1) the experience of living with COPD-related anxiety before participating in PR (e.g., “How did you experience COPD-related anxiety before participating in PR?”), (2) experiences during participating in PR in relation to the anxiety (e.g., “What did you learn/achieve from participating in PR…”), (3) the experience of living with COPD-related anxiety after participating (e.g., “How do you experience your anxiety after participating in PR?), and (4) additional comments (“Is there something you wish to add?”). Questions were generally open-ended and followed up by invitations to elaborate on individual experiences, for example, “Could you tell me more?” and “What does that mean to you?”

### 2.2 Data analysis

The Shapiro Wilk-test was used to test if data were normally distributed. Paired *t-*tests were applied to assess changes in all outcome variables from before to after PR. Change scores were computed for all outcome variables by deducting baseline scores from follow-up scores. Additionally, effect size estimates for each outcome was presented as Cohen's *d.* Associations between changes in COPD-related anxiety and changes in other PR outcomes were analysed in a series of correlation analyses. We did not use specific cut-points for descriptive categories of correlation coefficients, as they have been considered arbitrary and inconsistent (27), but we reported confidence intervals to inspect the range of plausible values of the coefficient. With the purpose of exploring missing data, all baseline variables for participants who did vs. did not complete the CAF-R questionnaire at follow-up were compared using *t*-tests and *chi^2^*-tests. The aforementioned analyses were conducted using Stata (28), with *p *< 0.05 as the level of significance.

Data analysis of the semi-structured interviews was inspired by the thematic analysis approach by Braun and Clarke (29). The analysis was conducted in six iterative phases. In phase 1, all transcriptions were read in depth and several times by the author EHB with the purpose of familiarizing herself with the data. In phase 2, all parts of the data that, directly or indirectly, referred to the experience of anxiety and/or management strategies were coded by EHB and labelled according to their contents. An inductive coding approach was applied at this stage, as the area has not previously been explored directly. In phase 3, multiple codes with similar contents were collated into potential themes by authors EHB and DS. In phase 4, EHB and DS reviewed the initial themes in relation to their individual codes and the essence of each theme was identified. In phase 5, the themes were labelled and, in phase 6, the themes were described and supported by patient quotations. The reporting of the themes was undertaken by EHB, DS and IFV. Interview quotations, selected to support the reporting of the thematic synthesis, were translated from Danish into English.

## 3 Results

### 3.1 Participant characteristics

**Figure 1:**
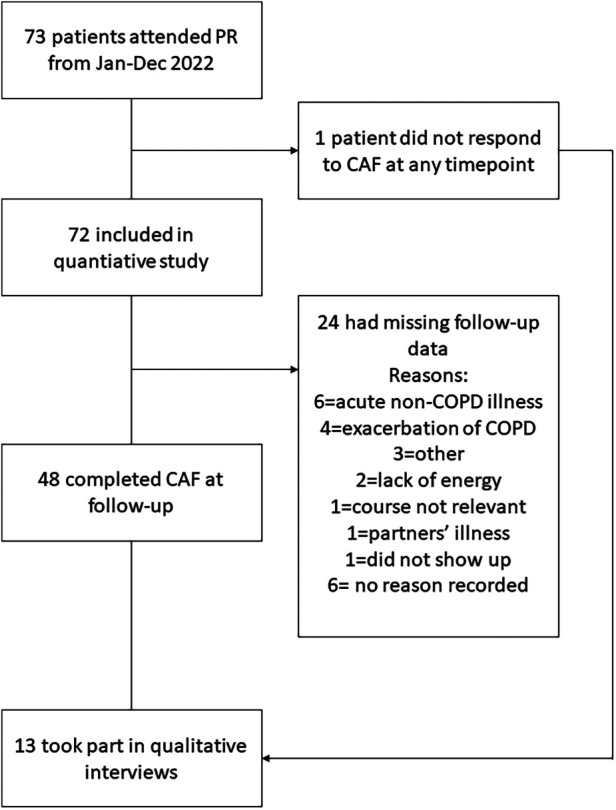
Participant flow chart.

A total of 72 patients with COPD were included in the quantitative study and 13 took part in qualitative interviews (see participant flowchart in Figure 1). An overview of participant characteristics of the total sample can be found in Table 1. An overview of characteristics of the interviewed patients can be found in Table 2. Patients who took part in interviews had a mean age of 68 (range: 52–80 years) and were predominantly female (*n* = 9, 70%). The interviewed patients had varying degrees of airflow obstruction, ranging from mild to very severe COPD. Interviewed patients reported baseline CAF-R scores from 3 to 54. Eight interviewed patients reported reduced CAF-R scores at follow-up, with change scores ranging from a 1-point to a 35-point reduction. Two interviewed patients reported increased CAF-R scores at follow-up, one of them representing a considerable increase from 18 to 40 points. Interviews had a mean duration of 29 min (range: 7–58 min).

**Table 1:** Participant characteristics.

	Total sample (*n* = 72)	Complete CAF-R data at follow-up (*n* = 48)	Missing CAF-R data at follow-up (*n* = 24)	*p* ^a^
Age, mean (SD)	71 (8)	71 (8)	69 (8)	0.25
Sex, *n* (%)				1.00
Male	34 (47%)	23 (48%)	11 (46%)	
Female	38 (53%)	25 (52%)	13 (54%)	
FEV1% pred., mean, (SD)	56.6 (19.7)	52.5 (17.6)	63.0 (21.6)	0.14
MRC score, mean (SD)	2.9 (0.9)	2.9 (0.7)	3.1 (1.2)	0.51
CAF-R, mean (SD)	24.1 (14.2)	24.4 (14.4)	23.5 (14.2)	0.80
CAT, mean (SD)	17.2 (6.5)	16.7 (6.2)	18.4 (7.3)	0.38
SF-12, mean (SD)				
Physical component score	36.4 (10.3)	38.3 (9.8)	31.0 (10.4)	0.019
Mental component score	42.0 (10.0)	44.1 (8.6)	36.3 (11.5)	0.009
6MWD meters, mean (SD)	388.3 (108.1)	409.4 (85.2)	339.0 (139.1)	0.014

6MWD, (6-minute walk distance); CAF-,R (COPD Angstfragebog revised, german for COPD Anxiety Questionnaire); CAT, (COPD Assessment Test); FEV1, (forced expiratory volume in first second); MRC, (Medical Research Council dyspnea score); SD, (standard deviation); SF-12, (12-Item Short-Form Health Survey).

^a^ The *p* value reflects the different between patients with complete vs. missing CAF data at follow-up.

**Table 2:** Characteristics of interviewed patients.

Interviewed patient number	Age	Sex	Degree of airflow limitation	CAF-R score at baseline	CAF-R score at follow-up
1	79	Male	Mild	43	12
2	66	Male	Mild	14	8
3	62	Female	NR	13	14
4	66	Female	Severe	42	NR
5	74	Female	Moderate	24	16
6	55	Male	Very severe	44	33
7	78	Female	Very severe	22	21
8	68	Female	Very severe	48	13
9	55	Female	Moderate	NR^a^	NR
10	66	Female	Moderate	18	40
11	76	Female	Severe	54	28
12	52	Female	Moderate	38	11
13	80	Male	NR	3	NR

CAF-R, (COPD Angstfragebog, German for the COPD Anxiety Questionnaire 20-item version); NR, (not reported).

^a^ The specific patient took part in the PR program and was interviewed but was missing CAF-R data at both time-points and was therefore not included in the quantitative analyses of the present study. Spirometry was performed outside of the PR setting prior to referral, and data was not transferred.

Patients who had missing CAF-R data at follow-up scored significantly lower on the SF-12 physical and mental components scores at baseline, compared to patients with complete CAF-R data (Table 1). Patients with missing data also presented with significantly lower walk distance (6MWD) at baseline, compared to patients with complete CAF-R data.

### 3.2 Changes in outcomes from before to after PR

Statistically significant improvements were seen across all outcomes from before to after PR, except for the CAT score, where the decrease in COPD-related disability did not reach statistical significance (Table 3). Change scores exceeded the MCID for the PCS of the SF-12, which was not the case for any of the remaining outcomes.

**Table 3:** Changes in outcomes from before to after PR in the total sample.

	Before PR		After PR		Change score	95% CIs	ES (*d*)
	Mean	SD	Mean	SD			
CAF-R	24.45	14.41	20.38	12.13	4.06	0.72–7.40	0.32
CAT	16.46	5.94	15.44	6.08	1.03	−0.59–2.64	0.17
SF-12							
PCS	38.05	9.51	42.45	8.53	−4.40	−6.76– −2.04	0.48
MCS	44.00	8.42	47.50	8.37	−3.50	−5.46– −1.54	0.41
6MWD	406.40	94.73	425.22	98.22	−18.82	−31.24– −6.39	0.19

6MWD, (6-minute walk distance); CAF-R, (COPD Angstfragebog revised, German for COPD Anxiety Questionnaire 20-item version); CAT, (COPD Assessment Test); CI, (confidence interval); ES, (effect size); MCS, (Mental component score); PCS, (Physical component score); PR, (pulmonary rehabilitation); SD, (standard deviation); SF-12, (12-Item Short-Form Health Survey).

### 3.3 Association between change in COPD-related anxiety and changes in other PR outcomes

Decrease in COPD-related anxiety from before to after PR was associated with increases in COPD-related disability, HRQoL, and functional exercise capacity (Table 4), but correlation coefficients between COPD-related disability and other PR outcomes were negligible.

**Table 4:** Correlation coefficients (*r*) between changes in COPD-related anxiety and changes in other outcomes from before to after PR.

	ΔCAF-R	ΔCAT	ΔSF-12 PCS	ΔSF-12 MCS	Δ6MWT
ΔCAF-R	1	−0.13 (−0.43−0.21)	−0.11 (−0.42–0.22)	−0.15 (−0.45–0.18)	−0.17 (−0.44–0.13)
ΔCAT		1	−0.41 (−0.65–−0.10)	−0.33 (−0.59–−0.003)	−0.07 (−0.38–0.25)
ΔSF-12 PCS			1	0.79 (0.63–0.88)	0.25 (−0.07–0.53)
ΔSF-12 MCS				1	0.32 (−0.00–0.57
Δ6MWD					1

6MWD, (6-minute walk distance); CAF-R, (COPD Angstfragebog revised, German for COPD Anxiety Questionnaire 20-item version); CAT, (COPD Assessment Test); MCS, (Mental component score); PCS, (Physical component score); PR, (pulmonary rehabilitation); SF-12, (12-Item Short-Form Health Survey). Confidence intervals (*r*) in parantheses.

### 3.4 Subjective experiences of changes in COPD-related anxiety during PR

**Figure 2:**
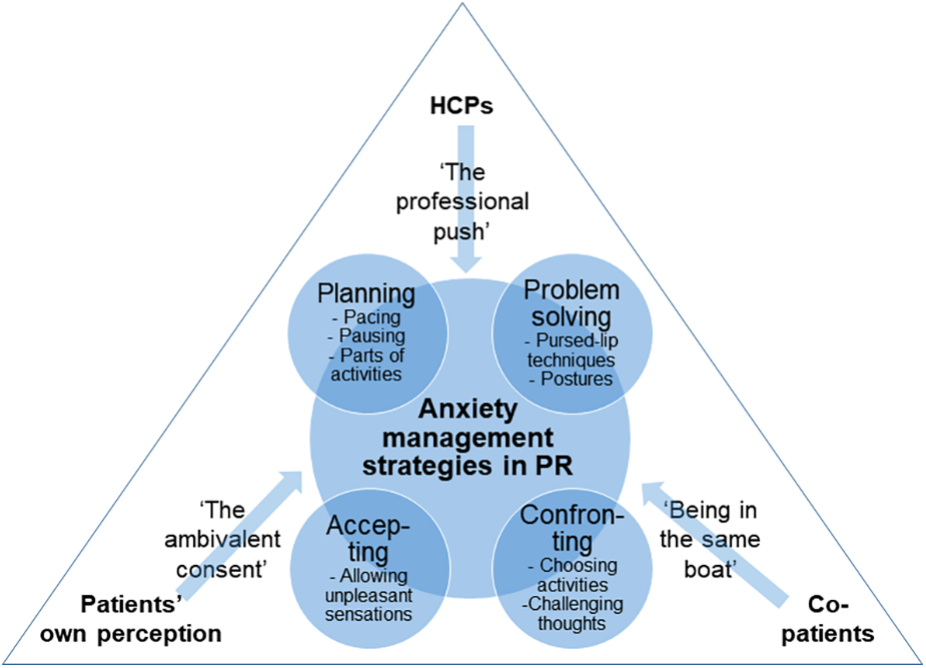
Anxiety management strategies and influential factors in pulmonary rehabilitation.

The qualitative analysis of patients’ narratives, after completing PR, regarding their experiences and gains from participation as well as the influential factors for perceived benefits, resulted in two main themes: (1) Anxiety management strategies, and (2) factors influencing the development of anxiety management strategies. Four types of anxiety management strategies were described, i.e., “planning”, “problem solving”, “confronting”, and “accepting”. The patients did not specifically label the strategies themselves, and patients were not explicitly instructed by HCPs in using these strategies. Rather, the strategies appeared to gradually and dynamically evolve over the course of the program, as patients gained new knowledge about their disease and were presented with activities, such as physical exercise and educational sessions. The development of the anxiety management strategies appeared to be influenced by three factors, i.e., “the professional push” from HCPs, “being in the same boat” with co-patients, and “the ambivalent consent”, which refers to patients’ perceived relevance and benefit of attending the program. A graphic overview of the themes and subthemes can be found in Figure 2.

#### 3.4.1 Strategies to manage COPD-related anxiety

##### 3.4.1.1 Planning

Patients described how feeling anxious or insecure about performing everyday life activities could lead to complete avoidance of activities:

*I don*’*t expose myself to stairs. I don*’*t expose myself to running. I don*’*t expose myself to anything physically demanding. Because I…well, I say it*’*s because I’m lazy… because I don*’*t feel like doing it…In some mysterious way, I just try to avoid it.* (Patient 2)

The planning strategy was described as an attempt to avoid everyday activities that would trigger breathlessness and associated anxiety. Participants described the strategy as a way of engaging in meaningful activities in everyday life, while, at the same time, preventing breathlessness and anxiety from spiraling out of control. As such, the strategy included careful planning of activities that patients knew would trigger breathlessness and anxiety. Planning involved balancing energy expenditure, for example by pacing and pausing the activities carefully. This strategy appeared to make patients feel a greater sense of control over their breathing and, thereby, also a greater control over feelings of anxiety.

*Well, I’m better at taking the breaks needed. I’ve been a bit of a bulldog, like, if you need to go shopping, then you’ll be used to doing that at a certain speed. One has these settled habits, if you will, about what one can usually do and how fast. And, it is a learning process for me to slow down. So, it*’*s about taking breaks, so that you, like, move from A to B more gently, rather than just going at full speed, because that works you up a bit, and then you need to stop to catch your breath* (Patient 6).

Planning could also involve the attempt to mentally break up everyday situations into smaller parts that felt easier to handle:

*What if I take the first nine steps and then stop. They used to have a chair, but then I did the nine steps, right leg first. I mean, I hear the physiotherapist*’*s voice in my head: “right leg first”. And that went well. Without ending up short-winded. I could feel it, of course, but I wasn't short-winded* (Patient 11).

Arranging “safety plans” prior to taking on an activity that could potentially lead to breathlessness and anxiety could also be part of the planning strategy.

*I can do much more than I could back then. Yes, and, of course, I always keep the phone close by when I go out. That makes me feel safer, should I get worse, I’ve got my phone, right* (Patient 8).

##### 3.4.1.2 Problem solving

In situations where an activity did lead to breathlessness and anxiety, patients described how they gradually learned to apply specific techniques and knowledge from the PR program to reduce uncomfortable breathing sensations and feelings. Hence, breathing techniques and posturing were described as helpful strategies to solve problems of breathing and anxiety in specific situations.

*If I’m having a very hard time breathing, I sit back in my chair and raise my arms above my head, and then I sit like that and stretch as much as I can. And then I breathe calmly, inhaling through my nose and exhaling through my mouth. And that has helped me. So, I really haven't had any fear of shortness of breath and I have not ended up in those really difficult situations* (Patient 8).

Patients also described how new knowledge about their disease, risk factors and medical inhaler devices influenced their treatment compliance and thereby gave them a sense of mastery over their symptoms.

*Because it [the course] has given me a lot of knowledge about what it [COPD] actually is. I do know that smoking, that I could change that, and they give you tools to do that. Now, you see, I have been taking asthma medicine for many years, and then I learn that I have been doing that all wrong* (Patient 10).

##### 3.4.1.3 Confronting

Over the course of the PR program, and linked in part to the problem-solving strategies, patients learned to actively confront situations they had previously thought would not be manageable.

*I’ve done that [confronted breathlessness], after I stopped in here, I’ve been going to the gym. And I started running. I’ve never been able to do that before in my whole life. I started running on the treadmill. And I can do that. I couldn't before. Now I can run, and I can keep running for longer and longer periods of time I mean, I’m getting in better shape* (Patient 12).

By choosing the situations that they wanted to confront, the patients gained a sense of control in spite of still feeling short of breath.

*I tested a breathing technique on myself. The sports hall has five floors, I went all the way up and down, up and down [the stairs], and I could feel that I was doing much better* (Patient 2).

Through their own experiences during activities in the PR program, patients learned that, although sensations of breathlessness can be unpleasant, they don’t have to be dangerous.

*The difference is huge. You get those tools for breathing, stretching your arms and stuff like that, right. And walking, getting out and moving around as much as possible. In the beginning, I walked to my mailbox and back, I was afraid to walk any further; what if I ended up breathless, right? Now I walk downtown and sometimes all the way down to Brugsen and Netto [local supermarkets]. Sure, I have to take a break now and again, but I get there and get back again, eventually* (Patient 8).

##### 3.4.1.4 Accepting

As patients gained new knowledge about physical activity, breathlessness, catastrophic thinking and anxiety during the PR program, they gradually became more familiar with situations that they would previously have avoided. This allowed for the evolvement of a more accepting strategy, which involved patients recognizing that they could not make unpleasant sensations go away, but that they did not have to be in the foreground of their lives and activities.

*Well, and then I’ve learned that I shouldn't be afraid of ending up short-winded. That it is not a problem. You won*’*t die or anything* (Patient 12).

*It doesn't preoccupy me so much, and, well, it did before. Back then, I was nearly sure that I would die whenever I experienced an episode* (Patient 8).

Accepting the symptoms, feelings and thoughts was not the same as “giving up”. Patients described how they actively started working on their thinking and attention, with the purpose of not letting unpleasant sensations and negative thoughts rule their lives.

*I’m not letting it [lung disease] take up too much space. It mustn't, and it mustn't bother me* (Patient 7).

#### 3.4.2 Factors influencing anxiety management strategies

##### 3.4.2.1 The professional push

“The professional push” is a subtheme that encompasses patients’ descriptions of how HCPs in the PR team contributed to their anxiety management by encouraging them to take part in physical exercise during the program, thereby also enabling them to, more generally, engage in meaningful everyday activities and the above-mentioned anxiety management strategies.

*Well, the kick in the butt, to be absolutely clear, that I received; It helped me. Maybe not so much physically, but mentally. The difference [between before and now] is so very, very, very, very big. Because I got back the courage to start walking again. I don*’*t do long walks or anything, I go out on the terrace to do some cutting and such. I’ve become a little bit more active. Before, I had become tied to my chair where I do my crocheting, knitting and watch the telly* (Patient 11).

The professional push was possible and beneficial when patients felt safe, seen, and recognized by the HCPs. These feelings were the prerequisites for patients accepting their push. This was expressed in various ways, for example when an activity was adjusted to fit the needs and preferences of individual patients:

*Immediately, when they realized that I wasn't making headway in there on machines, they had a chair for me and later also a couch, and then I learned how to sit down and how to get down on the floor and how to get back on my feet afterwards. So, it means a lot that the people in charge of the team [the Rehabilitation Team] say: “You need to train like this”* (Patient 11).

*We’re not handled in quite the same way. They take into account our different situations and the different stuff we’re grappling with* (Patient 6).

Contrastingly, PR was also described as a standardised package where all patients were offered the same interventions regardless of their individual needs and preferences. If certain patients felt they could not take part in certain activities, and if they felt there were no alternatives to that particular activity, it could impact their perceived outcome. For example, a patient experienced that there was no alternative to doing physical exercises in the fitness room, which made him panic and withdraw from the activity:

*I went down there [to the gym]. And I just stood there, trembling like a leaf. So, I walked over to the physiotherapist, something I wouldn't normally have done. And then I told him: “I’m not doing this”. And then he could see and hear from what I was saying that this simply wasn't going to work. I was completely panicking, I have never been in a situation like that before. It*’*s those bloody machines. He [the physiotherapist] said that he was Ok with that and then I went home* (Patient 3).

##### 3.4.2.2 Being in the same boat

The subtheme “Being in the same boat” refers to patients’ experiences of recognizing parts of themselves in the other PR participants and their feelings of fitting into the group of participants. Feeling equal, in terms of age, functional level and illness stage was described as a prerequisite for finding support in each other and learning from each other's experiences of managing difficult situations in the life with COPD.

*We exchange experiences and talk about how we’ve come to this and why it sometimes goes all wrong. So, it*’*s a way to move on a little* (Patient 1).

Feeling a part of the group was motivating, and the social aspect of the program was described as being as important to the outcome as the disease- and exercise-related aspects. On the other hand, the perceived outcome of the program could therefore also be compromised if patients felt they did not fit into the group.

*I’d very much like for them to set up a team where all participants were at the same level. And that we had nearly the same numbers [lung function], because then we’d be able to give each other more* (Patient 4).

Being able to recognize oneself in the other participants appeared to impact what patients chose to bring up in group discussions. Related to social acceptance, patients felt it was important that the topics they brought up resonated with the group. Patients who felt different from the rest of the group had difficulties opening up about topics, such as disease-related anxiety.

*When we’re there, all 14 of us, one might not want to share everything, for everyone to hear.” I mean, for those of us who have anxiety, and why that affects us and how you can get on top of it* (Patient 7).

##### 3.4.2.3 The ambivalent consent

Under the theme of “the ambivalent consent”, patients expressed varying levels of engaging in the PR activities depending on their perceptions of whether or not they did in fact have COPD, and whether or not they felt it was their own choice to be referred to the PR program in the first place.

*I’d like to start off by saying that I don*’*t believe that I have COPD. The people I know who have COPD, I can*’*t identify with them at all* (Patient 3)

*And then, I thought, OK, I best get started. Then, later I can joke with the doctor and tell him: “See, I don*’*t have that stuff [COPD] and now I’m above those 80% [lung function].* (Patient 12).

Readiness to take part in PR could be reduced in patients who did not acknowledge having COPD. Not accepting the diagnosis could create a feeling of ambivalence in relation to taking part in PR activities, which could be expressed by patients showing up for the group meetings, but not wanting to actively take part:

*All of that fitness and gymnastics and training, that*’*s not for me* (Patient 3).

The feeling of ambivalence could be strengthened if patients felt that they did not fit into the group (“In the same boat”) or if they did not feel recognized by the HCPs (“The professional push”). Ambivalent consent to taking part in the PR program could be strengthened if patients’ high expectations and motivation were not mirrored by HCPs’ attitudes.

*I asked my physician if there isn't anything I can do about it, some kind or training, for example, I bloody well need some training or something. But that was not an option, or so she said. I thought; “That just can*’*t be the case”. But that wasn't how things were done anymore. That was only done in the old days [pulmonary rehabilitation]. And then I thought: “Well”. But, then, my [spouse] went to the health center. [My spouse] talked to the people down there, and they said: “that*’*s a bloody lie, we’ve got hundreds of those courses”. So, I talked to the people at [the] Hospital, asking if they could do anything. And just one week later. I got a call from someone at the health center, saying that I could start training (Patient 6)*.

Moreover, if patients felt that the severity of the disease limited participation in PR activities, or if they did not feel that the activities had an effect at all, participation could be perceived as pointless:

*I’d hoped that the training would have an effect, but it*’*s a bit like: “Ah well, tough luck, and, like, you just can*’*t do shit about it”* (Patient 6).

## 4 Discussion

In the present study, systematic assessment of COPD-related anxiety was introduced in a standardized PR program. The results showed that average levels of COPD-related anxiety among PR participants decreased significantly from before to after the program, corresponding to a small effect size. This finding mirrors the already well-documented tendency for general anxiety levels to drop after PR (30). However, research suggests that a differentiation between general anxiety and disease-related anxiety is relevant in PR (15, 31). In a study by Reijnders et al. (17), CAF was used to assess COPD-related anxiety before and after a 3-week inpatient PR program. In line with the results of the present study, COPD-related anxiety was significantly reduced after PR. However, in the study by Reijnders et al., larger decreases in COPD-related anxiety were seen, as were greater improvements in other PR outcomes, i.e., COPD-related disability, HR-QoL and functional exercise capacity, even despite comparable baseline values in the Reijnders et al. sample and the present sample. Moreover, Reijnders et al. found a direct association between reductions in COPD-related anxiety and improvements in other PR outcomes, which was not demonstrated in our study. One possible explanation for this between-study incongruity may be related to differences in the contents, formats, and settings of the PR programs. In Reijnders et al., the program was delivered in an inpatient setting with up to seven sessions per week, and it included both group-based and optional individual psychological counselling. A dose-response effect may therefore have contributed to the differences in findings between our study and the study by Reijnders et al. There are not many studies of interventions specifically targeting disease-specific anxiety in COPD, but a meta-analysis (32) comparing high intensity cognitive behavioural therapy (CBT) in COPD to both low- (usual care without PR) and high-intensity (including PR) control conditions show that there is no significant effect in favour of CBT when compared to high-intensity controls, which may also, to some extent, be explained by the dose-response effect. Another possible explanation for the variant findings could be the relatively large proportion of missing follow-up data in our study, resulting in a relatively small sample size for the analyses. Also, it should be noted that a MCID for the CAF-R that was used to measure COPD-related anxiety in the present study has not been determined, and the clinical significance of the present results are therefore unclear.

Nonetheless, the qualitative results of the present study indicated that four types of strategies to manage disease-anxiety evolved over the course of the PR program. First, the “planning” strategy involved pacing, pausing, and dividing activities into smaller units. This strategy is an integral part of energy-enhancing interventions that are often delivered by occupational therapists in PR (33). However, to the best of our knowledge, its role in anxiety management in COPD has not been adequately described. Second, the “problem solving” strategy involved pursed-lip breathing techniques and postures to ease the airflow. These techniques are standard components in non-pharmacological breathlessness interventions, such as the Cambridge Breathlessness Intervention Service's Breathing, Thinking, Functioning-model (34), whose aim is to ameliorate catastrophic cognitions and dysfunctional breathing patterns. Third, the “accepting” strategy in the present study involved allowing oneself to engage in activities that might involve unpleasant breathing sensations, e.g., shortness of breath, instead of avoiding them or fighting against the sensations. This strategy aligns with a more meta-cognitive approach to anxiety management, where bodily sensations and accompanying thoughts are considered as fluctuating events that come and go automatically, and therefore do not require behavioral interference by the individual (35, 36). Fourth, the “confronting” strategy involved choosing to engage in meaningful activities in spite of potential shortness of breath and to challenge one's thoughts. Over time, associations between physical activity, breathlessness and anxiety may lead to avoidance of physical activity, and PR has been shown to act as a setting where catastrophic cognitions, behavioral habits and brain processing can be challenged and altered through assisted exercise and patient education (37, 38). Physical activity, through graded activity, that disconfirms fearful expectations has previously been shown to play an important role in the management of fear-related avoidance during PR (18). Exposure to feared activities and situations is an integral component in cognitive behavioral treatment of anxiety symptoms in general (39).

Hence, our qualitative results suggest that anxiety management strategies evolved gradually and dynamically over the course of the program, and they were described as being applied in a flexible manner where one strategy could be preceded or proceeded by another, depending on the situation. Across all strategies, the perception of being in control of the situation appeared to be important for anxiety management. This is in line with other studies, demonstrating that self-efficacy and COPD mastery, i.e., the confidence in one's ability to manage the chronic illness, are related to lower levels of anxiety (40).

Additionally, the qualitative results of the present study indicated that the anxiety management strategies were influenced by interactions with HCPs, co-patients, and the patients’ own perceptions of their situation over the course of the PR program. It should be noted that all three areas of influence could act either as facilitators of and barriers to anxiety management. For example, instructions from HCPs to engage in physical exercise (i.e., “the professional push”) could be perceived as an important way to engage in the “confronting” strategy. On the other hand, it could also be perceived as pressure to do something dangerous, which could lead to more avoidance and less anxiety management. Similarly, patients’ interactions with each other (i.e., “being in the same boat”) could lead to new ideas to management strategies, but the fear of not fitting into the group could also lead to further withdrawal from social interaction and support. Lastly, patients’ own perceptions of their situation could lead them to consent to participation in an intervention they did not think they needed (i.e., “the ambivalent consent”), which could result in surprising benefits, but also in a verification of the assumption that other people, in this case the external HCP who referred them to the rehabilitation program, did not understand their situation. To our knowledge, few studies exploring the impact of interpersonal aspects in COPD have been conducted, and many of them were outside the context of rehabilitation (41, 42). The available studies suggest that interactions between people with COPD and other individuals is associated with blame and negative affect, and there is a need to explore the role of interpersonal interactions in PR from multiple angles, with the aim of describing how anxiety management strategies can be optimally supported.

In spite of learning new anxiety management strategies, and in spite of a decrease in COPD-related anxiety from before to after rehabilitation, there was no direct association between changes in anxiety and improvements in other PR outcomes, which is somewhat surprising. Other factors may be the “missing link” explaining these improvements, for example increases in self-efficacy and -regulation, increased knowledge and motivation, or decreases in affective states (e.g., depression symptoms) (43), which were not addressed in the present study.

### 4.1 Implications for pulmonary rehabilitation practice

In the present study, standardized assessment of COPD-related anxiety was introduced in PR. The results showed that levels of disease-related anxiety decreased after the program, which may potentially be explained by the evolvement of anxiety management strategies during the program. Whether or not these management strategies are transferred to skills in daily management of anxiety is not clear, and should be explored more specifically in future studies. The CAF-R score that was used to assess COPD-related anxiety in the present study is relatively brief (20 min) and easy to administer in clinical practice. However, as a clinical cut-off has not yet been determined for the instrument, we cannot make any conclusions as to whether the reduction observed in the present study is clinically relevant. In the clinic, the CAF instrument should therefore always be followed up by a brief interview about the patient's response, e.g., including examples from everyday life.

Assessment of COPD-related anxiety can be included in clinical practice with the purpose of identifying an extra-pulmonary treatable trait that can be specifically targeted in PR (44, 45). However, measurement of disease-related anxiety in clinical practice should be accompanied by assessment of general anxiety, e.g., the Hospital Anxiety and Depression Scale, as low levels of disease-related anxiety do not necessarily rule out the presence of clinically significant general anxiety symptoms and/or an anxiety disorder. As we did not include measurement of general anxiety in the present study, we cannot conclude that the observed changes were *only* disease-specific and not explained by a generally high anxiety level and/or the presence of an anxiety disorder.

Approaches to targeting disease-related anxiety in the treatment of patients with pain has long been described and implemented in clinical practice (46). There is a need for similar approaches in COPD (44). The present study can be used to identify some of the factors in PR that could be included in an approach designed to target COPD-related anxiety in PR. When implementing initiatives to support anxiety management in COPD it is important to consider that interactions with HCPs during the program can act both as a facilitating and a hindering factor for the development of anxiety management strategies. The importance of HCPs’ responsiveness to patients’ help-seeking has also been described in a systematic review of qualitative studies of breathlessness management (47). Such responsiveness can be hindered by several factors, such as HCPs’ feeling under-resourced or ill-equipped, and it should also be noted, that some patients actively seek help for managing their symptoms, while others does not seek help until they are in acute crisis (47).

Upon completion of the present study, the health center management and HCPs decided to implement assessment of COPD-related anxiety as an integral part of their clinical practice. A cross-disciplinary panel followed the present project continuously during the study period and suggested that materials and instructions for assessing and addressing COPD-related anxiety during PR were made available for HCPs in other health centers in Denmark. Therefore, clinical assessment materials and instructions used in the present study are now available in Danish online (48).

### 4.2 Strengths and limitations

The present study is among the few existing studies that specifically address COPD-related anxiety in the context of PR. Data were collected in a real-world setting that adheres to the American Thoracic Society and European Respiratory Society guidelines for PR. A prospective design was applied in the quantitative evaluation, and qualitative interviews were conducted to yield insights into how patients managed their anxiety over the course of the program.

Nevertheless, a number of limitations should be mentioned. First, one-third of the included patients had missing CAF data at follow-up, and they reported significantly lower levels of HR-QoL and functional exercise capacity at baseline, compared to patients with complete data. This could suggest a bias in the analyses, as patients who were doing worse at baseline were not included. Second, and related to the first limitation, the sample in the quantitative analyses was relatively small, and several statistical tests were performed, which could increase the risk of Type I error. Third, we did not include longitudinal follow-up, and can therefore not conclude whether improvements in outcomes after PR were transferred into patients’ daily lives and maintained over time. Fourth, due to the real-world design of the study, we did not include a wait-list control group, and we can therefore not directly infer that the observed changes are caused by PR or by other confounding factors. Meanwhile, the qualitative results suggest that patients did indeed engage in anxiety management strategies influenced by factors in the PR setting. Also, the 6MWT was performed only once per patient at each measurement point, which might have resulted in increased risk of a learning effect and lower precision of 6MWD estimates (49, 50). Fifth, a measure of general anxiety was not included. Therefore, we cannot conclude that the observed changes were *only* disease-specific and not explained by a generally high anxiety level and/or a high prevalence of anxiety disorders in the sample. Lastly, the person responsible for the selection of the patients for interviews in the present study was also in charge of delivering the PR program. The selection of patients might therefore be influenced by other (informal) criteria than they ones selected for purposive sampling *a priori*.

## 5 Conclusion

The quantitative results of the present study showed a significant decrease in COPD-related anxiety from before to after PR. We found significant increases in HR-QoL and functional exercise capacity, but these changes were not directly associated with the change in COPD-related anxiety. The qualitative analysis identified four anxiety management strategies: “planning”, “problem-solving”, “accepting” and “confronting”. These management strategies appeared to be influenced by interactions with HCPs, interactions with co-patients and the patients’ own perceptions of their situation.

## Data Availability

The raw data supporting the conclusions of this article will be made available by the authors, without undue reservation.
